# Structural characterization and magnetic response of poly(*p*-xylylene)–MnSb and MnSb films deposited at cryogenic temperature

**DOI:** 10.1038/s41598-021-95475-9

**Published:** 2021-08-06

**Authors:** L. N. Oveshnikov, S. A. Zav’yalov, I. N. Trunkin, D. R. Streltsov, N. K. Chumakov, P. V. Dmitryakov, G. V. Prutskov, O. A. Kondratev, A. A. Nesmelov, S. N. Chvalun

**Affiliations:** 1 National Research Center “Kurchatov Institute”, Moscow, 123182 Russia; 2 N.S. Enikolopov Institute of Synthetic Polymeric Materials, RAS, Moscow, 117393 Russia

**Keywords:** Materials for devices, Magnetic properties and materials, Organic-inorganic nanostructures

## Abstract

In this study, we employed several experimental techniques to investigate structure and magnetic properties of poly(*p*-xylylene)–MnSb composites synthesized by low-temperature vapor deposition polymerization technique and MnSb films deposited at various temperatures. The presence of MnSb nanocrystallites in the studied films was verified by the results of X-ray diffraction, electron microscopy and Raman spectroscopy studies. The obtained data revealed the formation of Sb-rich sublayer with well-oriented Sb grains near the susbtrate, which seems to act as a buffer for the consequent poly(*p*-xylylene)–MnSb or MnSb layer growth. Increasing the polymer content results in qualitative change of surface morphology of studied films. At high polymer content the hybrid nanocomposite with MnSb nanoparticles embedded into poly(*p*-xylylene) matrix is formed. All investigated samples demonstrated detectable ferromagnetic response at room temperature, while the parameters of this response revealed a complex correlation with nominal composition, presented crystal phases and surface morphology of studied films. Estimated values of the Curie temperature of the samples are close to that of bulk MnSb.

## Introduction

Nowadays, composite materials are widely used for various applications^1^. Great interest in such materials stems from the possibility to utilize different functional properties within one system, which helps to substantially increase the efficiency of a resultant device or material. Classically, the matrix material determines basic properties of a composite system, while inclusions of the filler can modify these properties or introduce new ones. When characteristic sizes of the filler inclusions fall down to nano-scale, i.e. nanocomposite is formed, size-effects become important and may substantially alter the properties of the system, including both, the properties of the components and the interplay between them. It is well-known that size-effects determine mechanical^2,3^, electronic^4–6^, optical^7,8^ and magnetic^9^ properties of the nanodots and nanoparticles. Thus, nanostructuring becomes one of the key approaches of engineering the properties of various systems required for applications (e.g. tuning the spectral range of photodetectors or the disorder level in thermoelectric materials). In particular, recently the studies of nanostructured magnetic systems have gained renewed interest due to perspectives of magnetophotonic applications^10^. Moreover, magnetic nanocomposites represent a fruitful platform for realization of various spintronic devices^11^. The latter, basically, requires spin-polarization of electrical current, which usually is induced by ferromagnetic (FM) subsystem within the material. In this respect, the key advantage of magnetic nanocomposites is the possibility to obtain FM state with Curie temperature, $T_c$, substantially above the room temperature, while more “classical” systems in this field, namely diluted magnetic semiconductors, yield much lower $T_c$ values (e.g. the highest $T_c$ values for widely studied (Ga,Mn)As systems are below − 80 °C^12,13^). While $T_c$ value for a nanocomposite is mainly determined by the FM material, which usually serves as a filler, another key requirement for spintronic applications is the presence of interaction between conducting and magnetic subsystems. The latter depends on the combination of properties of matrix and filler materials. Among various magnetic nanocomposites, many promising results were reported for systems based on a III-V semiconducting matrix with MnSb inclusions^14–19^. Aside from high-$T_c$ FM phase, for GaSb–MnSb composites the anomalous Hall effect^20^ was observed at room temperature ^15,19^, which demonstrates the presence of interaction between conducting and magnetic subsystems, i.e. the appearance of spin-polarized current required for spintronic applications. However, for the InSb–MnSb system, with smaller band-gap of the matrix material (InSb), such interaction was damped due to the formation of Schottky barriers on the boundaries of MnSb inclusions^16^. Thus, using material with wider band-gap as a matrix can lead to an increase of carrier spin-polarization, while achieving the hopping transport regime can also increase the amplitude of related phenomena (by an analogy with tunnel magnetoresistance effect) and of response to the external stimuli (e.g. light). Therefore, corresponding nanocomposite system can become an efficient material for spintronic devices.

Hybrid nanocomposites (i.e. polymer matrix with inorganic filler) represent relatively new research field. This type of systems have been intensively studied in recent years as they incorporate several new features as compared to typical inorganic composites. In particular, using poly(*p*-xylylene) (PPX) as a matrix provides the possibility to obtain flexible and bio-compatible nanocomposites. Various PPX-based nanocomposites with metallic^21,22^, semiconducting^23,24^ and magnetic^25,26^ fillers were successfully synthesized using low-temperature vapor deposition polymerization (VDP) method^27^. Aside from primary properties variations, such nanocomposites are promising for applications due to their gas-sensing^28^ and memristive^29^ properties, while magnetic hybrid nanocomposites also can be used for spintronic applications. In comparison with inorganic composites, hybrid nanocomposites provide the possibility to use elastic deformation of polymer matrix as an additional variable parameter to tune/control the properties of such systems or to create tactile sensors. Aside from that, polymer matrix serves for stabilization of filler nanoparticles, preventing their agglomeration, and provides the possibility to substantially alter their sizes and distances between them (by varying the synthesis conditions), which is required for actual applications. In particular, incorporating MnSb inclusions into the PPX matrix (which is, essentially, a wide band-gap insulator) may become an efficient way to obtain high-$T_c$ FM nanocomposite with high degree of carrier spin polarization. In this respect, the advantage of MnSb filler is that, unlike some elemental FM metals (such as Fe^26^), the properties of corresponding inclusions in the PPX matrix should be affected by the unintended oxidation processes in a rather predictable manner. In particular, bulk Mn oxides are paramagnetic at room temperature, thus, oxidation should simply decrease the volume of MnSb phase that is FM at room temperature. Studies of the effects of surface oxidation of MnSb films showed that the thickness of such oxide layer is about 4–7 nm^30^. Which is why the overall decrease of magnetization due to surface oxidation is negligible for sufficiently thick (e.g. 200 nm) films. It should be noted that the presence of surface oxide layer may increase the coercive force of the MnSb film due to locally randomized exchange bias phenomenon^30^. Low-temperature VDP route implies the deposition on the substrate held at cryogenic temperatures, which may lead to the formation of nanoinclusions with distorted crystal structure or even molecular clusters^24^, affecting the resultant properties of the nanocomposite. At cryogenic temperatures the formation of binary phase inclusions (e.g. MnSb) from elemental precursors seems to have rather low probability, which is why for VDP synthesis binary precursors seem to be more suitable^23,24^. However, for MnSb the effects of such deposition conditions, let alone the incorporation into organic matrix, have not been studied in detail yet.

Bulk MnSb is a FM semimetal with $T_c \approx 300{-}320$ °C^31,32^ and room temperature saturation magnetization $M_S \approx 700$ emu/cm$^{3}$^33,34^. The increase of Mn atoms excess in the Mn$_{1+\delta }$Sb compound leads to the considerable decrease of $T_c$ and $M_S$ values^31,32^. Due to compatibility with conventional III-V semiconducting materials, thin films of pure MnSb have been intensively studied as a perspective material for creation of efficient FM/semiconductor heterostructures. In relevant works, MnSb films were usually obtained from the elemental sources using molecular-beam^35–40^ and hot-wall^41–44^ epitaxy techniques. As an alternative, several groups studied films obtained via layer-by-layer Mn-Sb deposition. For Mn-Sb superlattices, it has been shown that binary MnSb phase can be formed even at low temperatures (− 100 °C), when the thickness of deposited layers are about several nanometers^45^, while for thicker bilayer films post-growth annealing is required to obtain detectable MnSb phase^46^. There were few reports on synthesis of MnSb films from a binary precursor using pulsed laser deposition^47^ and magnetron sputtering^48^. In addition, the MnSb nanostructures were studied in a form of netlike nanocrystals^49^, and spherical nanoparticles^50–52^. Reported results suggest that the FM response parameters of corresponding MnSb-based systems severely depend on the chosen synthesis method, while even for a single method, resultant tendencies are rather questionable. For example, for MnSb films, obtained using hot-wall epitaxy technique, an increase of crystallinity, induced by an increase of substrate temperature^43^, results in an increase of coercive force, $H_c$, and a decrease of $M_S$ values. Thinner MnSb films reveal small compressive strain of crystal structure, which becomes negligible as the film thickness (deposition time) is increased^42,44^. Different groups reported that increased deposition time result in an increase^44^ or a decrease^42^ of mean crystallite size, while in both cases it leads to a decrease of $H_c$ and an increase of $M_S$ values. Moreover, the $M_{R}/M_{S}$ ratio ($M_{R}$ is the remanent magnetization value) for corresponding films shows opposite tendencies as a function of $H_c$^42,43^. Thus, the inverse relation of $H_c$ and $M_S$ seems to be the only universality (within a single sample series) observed for MnSb-based systems^50^, while the absolute values may substantially vary. Therefore, it is crucial to verify that in the framework of chosen synthesis method the formation of composite system is not accompanied by the substantial degradation of MnSb phase and by corresponding loss of ferromagnetic properties of the system. Furthermore, to obtain MnSb-based nanocomposites with optimal parameters one needs to know basic tendencies of structural features and magnetic properties of studied systems as a function of synthesis conditions.

In this work, we employ several experimental techniques to investigate structure and magnetic properties of MnSb and PPX–MnSb films in order to elaborate basic tendencies related to the low-temperature deposition and addition of organic component. It is well known that the decrease of size of magnetic nanoparticles eventually leads to the transition from the FM into superparamagnetic state^9^. Usually, the increase of PPX content in a composite leads to the decrease of size of filler inclusions^23,24^, which is why, in order to retain FM state we investigated systems with relatively low PPX content. However, even within studied range of compositions we observed successful formation of PPX–MnSb nanocomposite system that demonstrate pronounced FM at room temperature, i.e. suitable for applications.

## Results and discussion

Composite poly(*p*-xylylene)–MnSb (PPX–MnSb) films were obtained using low-temperature VDP technique^27^ on (100)-oriented Si substrates. In such process, the monomer and filler vapors are adsorbed simultaneously on a substrate, cooled with liquid nitrogen ($T_{sub} = -196$ °C). The intensity of monomer vapor flow depends on the sublimation temperature $T_{\mathrm {PX}}$ of the monomer (*p*-xylylene–PX) precursor. The filler precursor (MnSb) was evaporated from a tantalum boat, heated with applied current $I_{\mathrm {MnSb}}$. Studied composite samples were obtained using different $T_{\mathrm {PX}}$ values, while $I_{\mathrm {MnSb}}$ was held constant (i.e. constant filler vapor flow intensity). After deposition step (deposition time for all studied samples was around 180 min) the substrate was slowly heated up to room temperature, resulting in polymerization of PX and formation of MnSb inclusions. The reference samples MS-RT, MS-LT and MS-LT* were obtained without sublimation of the monomer precursor. Samples MS-LT and MS-LT* were deposited on cooled substrates ($T_{sub} = -196$ °C), while the MS-RT sample was deposited on a substrate held at room temperature ($T_{sub} = 20$ °C). In addition, the sample MS-LT* was obtained at higher rates of MnSb precursor evaporation (higher $I_{\mathrm {MnSb}}$ value). Basic parameters of the studied films are summarized in Table 1.

**Table 1:** Sample parameters: substrate temperature $T_{sub}$; sublimation temperature of the monomer precursor $T_{\mathrm {PX}}$; electrical current $I_{\mathrm {MnSb}}$ applied to the tantalum boat for the MnSb evaporation; film thickness *d*; mean size of MnSb crystallites $D_{Sch} [\mathrm {MnSb}]$; RMS roughness $R_q$; coercive force $H_c$, remanent magnetization $M_{R}$, saturation magnetization $M_{S}$ and corresponding $M_{R}/M_{S}$ ratio at room temperature for studied samples.

Sample	$T_{sub}$ ($^\circ$C)	$T_{\mathrm {PX}}$ ($^\circ$C)	$I_{\mathrm {MnSb}}$ (A)	*d* (nm)	$D_{Sch} [\mathrm {MnSb}]$ (nm)	$R_q$ (nm)	$H_c$ (Oe)	$M_{R}$ (emu/cm$^{3}$)	$M_{S}$ (emu/cm$^{3}$)	$M_{R}/M_{S}$
MS-RT	20	–	13	288	34	2.9	303	303	466	0.65
MS-LT	− 196	–	13	315	16	3.1	236	113	241	0.47
MS-LT*	− 196	–	14	303	16	2.1	95	36	93	0.39
MS-60	− 196	60	13	308	20	2.5	164	116	285	0.41
MS-70	− 196	70	13	260	21	2.8	206	201	271	0.74
MS-90	− 196	90	13	175	8	5.5	215	15	27	0.55
MS-100	− 196	100	13	240	13	6.3	246	124	186	0.67

**Figure 1:**
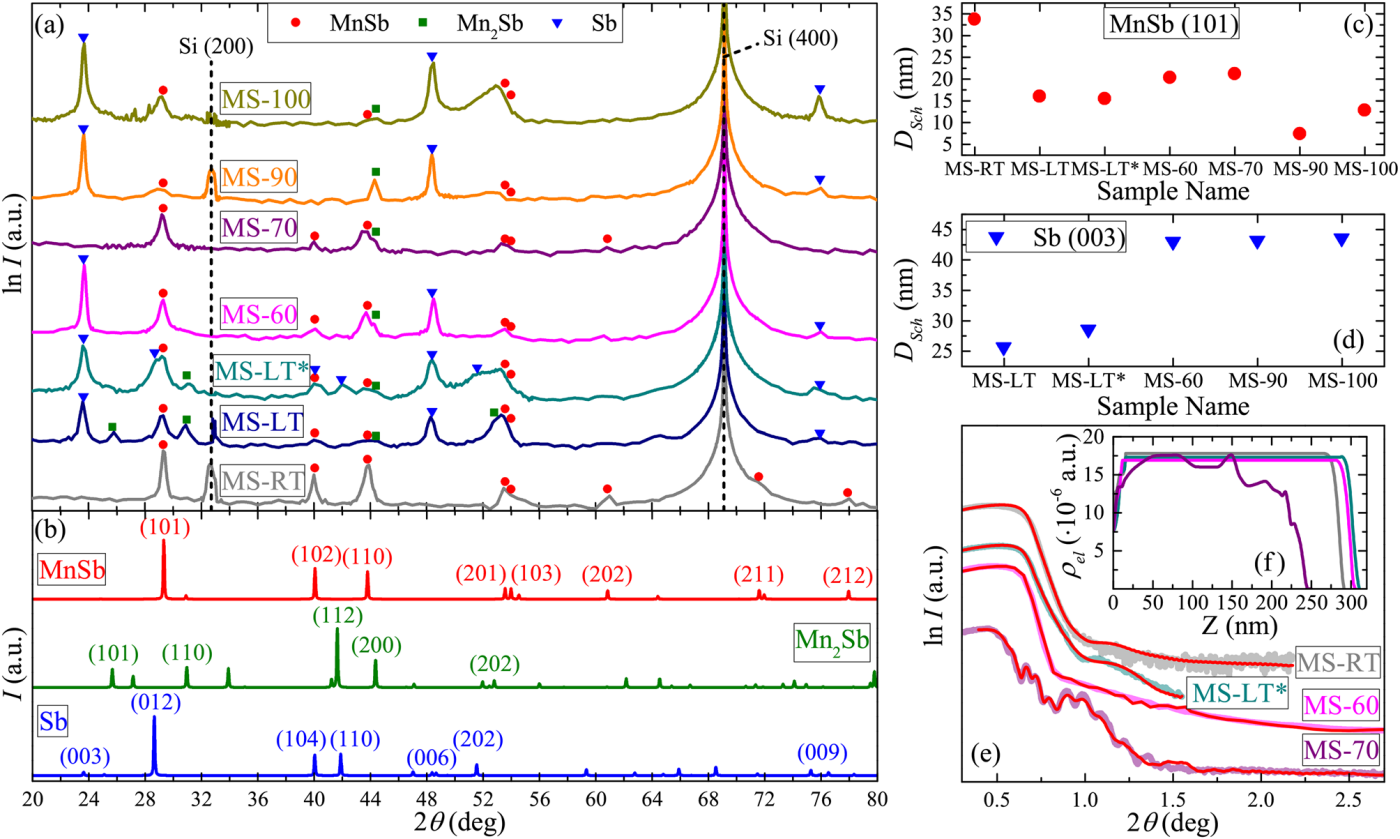
(**a**) Experimental X-ray diffraction patterns of studied films in logarithmic scale, curves are vertically shifted for clarity. Symbols in this panel correspond to the peak positions of reference powder XRD patterns for pure MnSb (PDF-2 01-072-5844), Mn$_2$Sb (PDF-2 01-089-4962) and Sb (PDF-2 01-085-1322). Corresponding patterns with marked *hkl* indices of considered reflections are given in panel (**b**), in linear scale. Mean crystallite size for studied films estimated from (**c**) MnSb (101) and (**d**) Sb (003) reflections using Scherrer equation (1). (**e**) Experimental X-ray reflectivity curves for several studied samples, curves are vertically shifted for clarity. Corresponding fitting curves are shown as red lines. Obtained electron density profiles as a function of distance from the substrate are given in (**f**).

The X-ray diffraction (XRD) patterns of investigated films were studied in the range of $2\theta = 20^\circ$–$80^\circ$. Corresponding patterns are presented in Fig. 1a in logarithmic scale. For all studied films we observed strong Si (400) reflection, related to the substrate, and for several samples we also observed low-intensity peaks at the angle position of the forbidden Si (200) reflection. These peaks appear due to the multiple diffraction phenomenon, and are often accompanied with additional peculiarities, such as broad shoulders or subpeaks^53^, which are also present in the XRD patterns (Fig. 1a). Aside from peaks related to the substrate, obtained XRD patterns can be reasonably interpreted in terms of three crystalline phases: hexagonal MnSb (s.g. $P6_3/mmc$), tetragonal Mn$_2$Sb (s.g. *P*4/*nmm*) and trigonal Sb (s.g. $R \bar{3}m$). Reference powder XRD patterns of these materials are shown in Fig. 1b. The results of phase analysis of experimental XRD patterns are shown in the Fig. 1a by various symbols, corresponding to suitable reference peak positions. The *hkl* indices of considered reflections in reference patterns are also marked in Fig. 1b.

The XRD pattern for the reference MS-RT sample (Fig. 1a) shows several peaks of the MnSb phase with relative intensities close to that of the powder reference, suggesting arbitrary orientation of crystalline grains. Similar pattern, but with lower intensities, can be observed for other samples, implying the presence of MnSb crystallites in all studied films. However, for samples with high polymer content (MS-90 and MS-100) one can reliably distinguish only the most intensive MnSb (101) peak, while the experimental peak is slightly shifted towards lower $2\theta$ values, in comparison with other samples, suggesting possible tensile strain of MnSb nanocrystallites in these films. It also should be noted that for several samples one can observe a wide peak near $2\theta = 53^\circ$, which may include several MnSb reflections, however, relatively high intensity of this peak (especially for the samples MS-LT, MS-LT* and MS-100) suggests possible appearance of preferable orientation or shape anisotropy of MnSb nanocrystallites. To estimate mean crystallite size we used Scherrer equation:

1 $$\begin{aligned} D_{Sch}=K\lambda _{X}/(\beta _{hkl} \mathrm {cos}~\theta _{hkl} ) , \end{aligned}$$

with $K=2 \sqrt{\mathrm {ln}2/\pi }$ and $\lambda _{X} = 1.5406$ Å ($\beta _{hkl}$ and $\theta _{hkl}$ are the FWHM and position of the reflection, correspondingly). The $\beta _{hkl}$ values were estimated using fitting of corresponding XRD peak with pseudo-Voigt function. The estimated $D_{Sch}$ values corresponding to the MnSb (101) peak for studied samples are shown in Fig. 1c and listed in the Table 1. As one can see, the mean size of MnSb crystallites for the MS-RT sample ($T_{sub} = 20$ $^\circ$C) is substantially higher in comparison with the films deposited on a cooled substrate ($T_{sub} = -196$ $^\circ$C). Corresponding samples show similar $D_{Sch}$ values at low (or zero) polymer content, while at higher polymer content $D_{Sch}$ decreases further. The minimum $D_{Sch}$ value, below 10 nm, is observed for the sample MS-90.

Several peaks in the presented XRD patterns can be attributed to the appearance of the Mn$_2$Sb phase. However, obtained results suggest that corresponding crystallites have preferable orientation relative to the substrate plane. This conclusion stems from the fact that XRD patterns for studied films do not include any traces of the most intensive Mn$_2$Sb (112) reflection of the powder reference, while almost all patterns include possible traces of the Mn$_2$Sb (200) reflection. The samples MS-LT and MS-LT* also reveal additional Mn$_2$Sb (110) reflection, which yields $D_{Sch}$ values of about 12–15 nm. For the MS-LT sample one can also observe the weak Mn$_2$Sb (101) reflection, suggesting that wide peak near $2\theta = 53^\circ$ may also include the Mn$_2$Sb (202) reflection. However, for other samples it is unlikely that this peak can be related to the Mn$_2$Sb phase, due to the absence of any traces of the first-order Mn$_2$Sb (101) reflection.

One of the key problems in synthesis of MnSb thin films^40,54^ and nanoparticles^51,52^ is partial segregation of Sb. Given the powder-like intensity profile of MnSb phase in our samples, one can assume that potentially segregated Sb crystallites should also yield peaks with relative intensities close to those of the powder reference, suggesting the absence of preferred crystal orientation. However, in our case for several samples one can observe pronounced peaks related to the Sb (003) (006) and (009) reflections, suggesting the appearance of well-oriented Sb crystallites. The presence of non-oriented Sb phase is observed only for the MS-LT* sample, which suggests that the increase of the temperature of MnSb precursor evaporation leads to different regime of sample growth. Presumably, such effect can arise due to higher fraction of elemental Sb in the vapor. However, due to the absence of reliable data on temperature dependence of vapor composition for MnSb, to verify our assumption additional studies are required, which are out of the scope of this paper. For this sample mean crystallite size estimation for the Sb (012) peak (superimposed on the MnSb (101) reflection) gives the $D_{Sch}$ value of about 10 nm. As for oriented Sb phase, similar estimation using the Sb (003) peak yields much higher $D_{Sch}$ values, presented in Fig. 1d. As one can see, the presence of monomer flow results in an increase of $D_{Sch} [\mathrm {Sb (003)}]$ value. Taking into account pronounced orientation of crystallites and rather limited effect of the deposition conditions (i.e. the $T_{\mathrm {PX}}$ values) on the $D_{Sch}$ values for this phase, one can assume that the (003)-oriented Sb crystallites should form at the beginning of the depositions process, i.e. near the substrate, which was confirmed by the results of electron microscopy studies discussed below. It should be noted that the XRD patterns show no distinguishable traces of Mn and/or Sb oxides.

Figure 1e shows the X-ray reflectivity (XRR) curves, obtained for several samples. These experimental curves were fitted using the Abel matrix formalism and calculated electron density profiles are presented in Fig. 1f. As one can see, the Kiessig fringes in the experimental XRR curves can be resolved only for the MS-70 sample. The absence of Kiessig fringes can be related to considerable blurring of boundaries and relatively large thickness of studied films. The latter is valid in case of small variation of optical constants across the layer, suggesting that studied films are homogeneous. Thus, to obtain electron density profiles for the investigated samples we relied on the film thickness values *d* estimated from the electron microscopy data (see Table 1). For the MS-70 sample fitting procedure yields rather complex electron density profile suggesting multilayered structure of the film. Estimated mean density values for the samples MS-RT, MS-LT and MS-60 are 6.42, 6.11 and 6.00 g/cm$^3$ correspondingly. These values appear to be lower than the density values of MnSb (6.90 g/cm$^3$) and Sb (6.69 g/cm$^3$), which can be related to highly-disordered structure of studied films. These films show certain density decrease near the substrate, while for the sample MS-70 one can observe substantial decrease of density in the upper sublayer, with estimated mean density value of about 4.93 g/cm$^3$. Using estimated density for the MS-RT sample (6.42 g/cm$^3$) as a baseline and typical value of PPX density (1.10 g/cm$^3$) one can estimate maximum PPX content in this sublayer as about 28 vol$\%$, while for the MS-60 sample similar estimation yields integral PPX content of about 8 vol$\%$, which is in agreement with the difference in deposition conditions and one can suggest incorporation of the polymer into the film.

**Figure 2:**
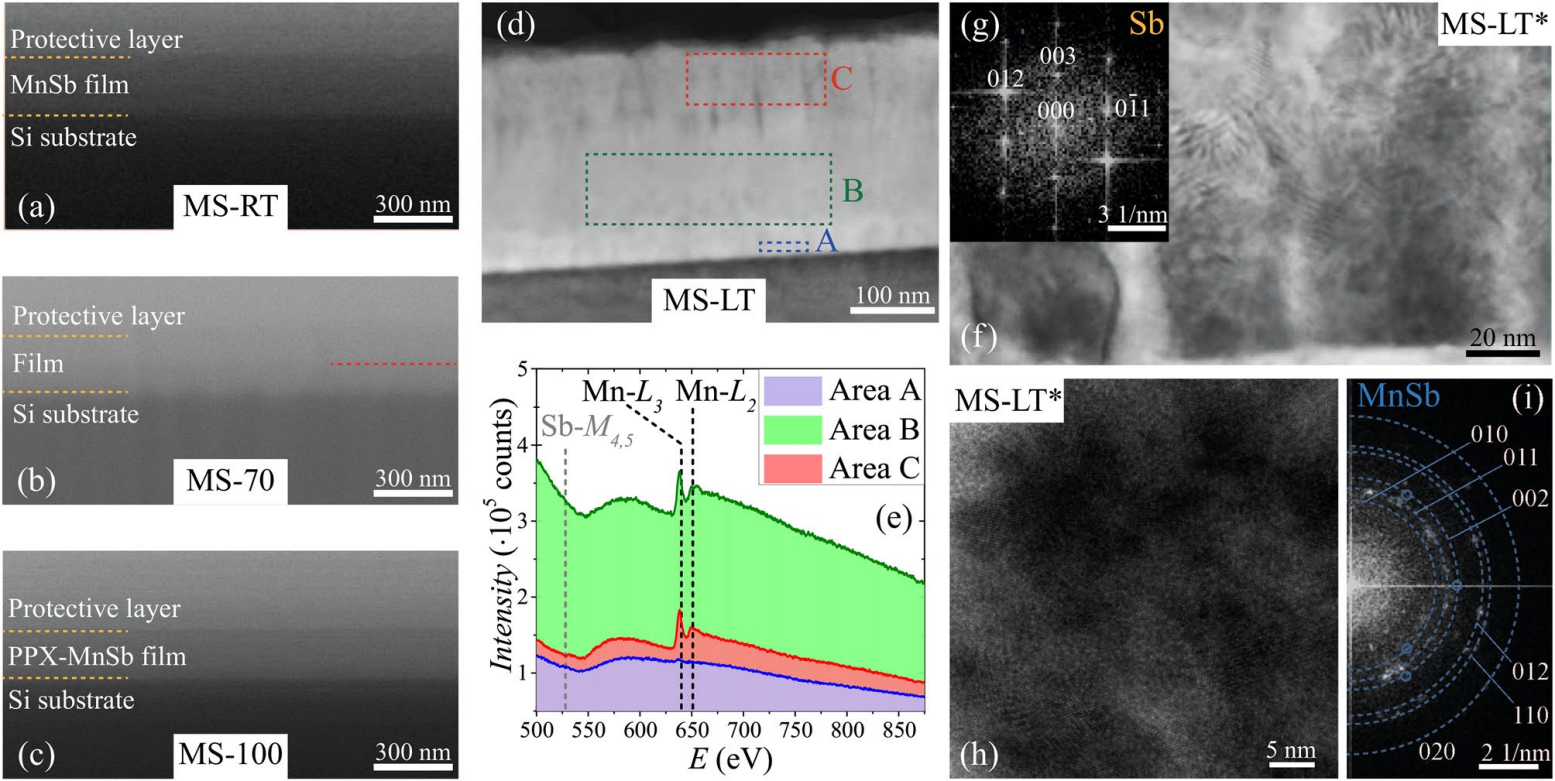
SEM images of films edges, obtained by the ion beam etching, for the samples (**a**) MS-RT, (**b**) MS-70 and (**c**) MS-100. To visualize the edge, the samples were tilted. Yellow dashed lines mark the interfaces of the films with the substrate and protective W layer. Red dashed line in panel (**b**) marks the position of sublayer for the sample MS-70 (see text). (**d**) STEM HAADF cross-section image for the sample MS-LT. Colored frames represent EELS scanning areas in different parts of the film. (**e**) Corresponding EEL spectra for different scanning areas with marked positions of ionization edges for Mn and Sb. (**f**) Cross-section TEM image for the bottom part of the MS-LT* film, close to the substrate. Darker regions correspond to oriented Sb grains. (**g**) Fast Fourier transform pattern of such Sb grain with indexed reflexes. (**h**) Cross-section TEM image for the upper part of the MS-LT* film. (**i**) Fast Fourier transform pattern of the image in (**h**). Dashed circles correspond to several interplanar distances characteristic for the MnSb. Weak reflexes are additionally marked with small circles. Presented SEM and TEM/STEM images were processed using digital micrograph software (Gatan) and TEM Imaging&Analysis software (ThermoFisher Scientific).

In general, the thickness of PPX-based composite films depends not only on nominal PX/filler flow rates, but also on sticking coefficients of molecules of each flow, which can depend in a complex manner on flow intensities. Thus, the thickness of studied films was estimated independently, using the scanning electron microscopy (SEM) results. For this purpose investigated films were locally etched with Ga$^+$ ions, to obtain clear film edge. Typical SEM images of such film edges are presented in Fig. 2a–c. With the exception of the MS-70 film, all studied films reveal homogeneous contrast of the film region, which can be distinguished from the Si substrate and W protective layer. This observation suggest the absence of relatively large-scale inhomogenties within the film. Analogous image for the MS-70 sample (Fig. 2b) shows the presence of additional layer (seen as a darker stripe, marked with red dashed line in Fig. 2b). The presence of this layer agrees well with the density profile for this film obtained from the XRR curve fitting and discussed above. Obtained density profiles for other films are also in agreement with the homogeneous contrast of corresponding SEM images. The resultant values of integral film thickness, *d*, determined from SEM images are listed in Table 1.

The samples MS-LT and MS-LT* were additionally studied by transmission electron microscopy (TEM). Both samples revealed similar peculiarities of microstructure. The integral film thickness for these samples estimated from TEM measurements is in a good agreement with the values obtained from SEM images. Figure 2d shows the scanning TEM (STEM) image of the sample MS-LT cross-section, obtained using a high-angle annular dark-field (HAADF) detector. To visualize possible variations of elemental composition we performed electron energy loss spectroscopy (EELS) scanning over several regions of the film, marked by colored frames in Fig. 2d. The corresponding EEL spectra are shown in Fig. 2e. All EEL spectra contain wide peak in 548–630 eV region, which is characteristic for Sb. However, double peak in the 640–650 eV region, characteristic for Mn atoms, is almost absent in the spectrum obtained from the area A (near the substrate). In the EEL spectra obtained for areas B and C, i.e. recorded from the upper parts of studied film, one can see well-pronounced Mn-related peaks. Thus, obtained EELS results suggest that Sb-rich buffer sublayer is formed at the beginning of the deposition process, above which the binary Mn-Sb layer is grown. Closer inspection of the bottom sublayer of the MS-LT* film (corresponding TEM image is presented in Fig. 2f) reveals the presence of darker grains. The fast Fourier transform (FFT) pattern of a grain, shown in Fig. 2g, reveals well-pronounced reflexes, corresponding to the Sb crystal. Moreover, the FFT patterns for several of these Sb grains shows that [003] crystal direction is almost perpendicular to the substrate plane. Thus, the observed (003*l*) reflections in the XRD data can be attributed to well-oriented Sb grains, formed in the bottom Sb-rich sublayer of the films. Closer inspection of TEM images of the upper part of the MS-LT* film (typical TEM image is shown in Fig. 2h) reveals the presence of small areas with distinguishable atomic layers, suggesting the presence of large number of small crystallites, which agrees well with relatively low intensity of the XRD patterns. However, the FFT pattern of this image, shown in Fig. 2i, reveals numerous reflexes, corresponding to the interplanar distances in the MnSb crystal, which is also in agreement with the XRD results.

The appearance of the Sb-rich buffer sublayer can be related to the incongruent character of MnSb melting^46^, implying the possibility of elemental (Mn and Sb) vapors formation. Because Sb has higher volatility than Mn, Mn-Sb flow can be non-stoichiometric at the early stages of evaporation. As for oriented-Sb phase, the preferable (003)-orientation was reported for large ($\sim 100$ μm) Sb grains deposited in a hot-wall reactor on glass substrates^55^. Partial (003)-orientation, deduced from corresponding redistribution of XRD peak intensities (as compared to powder XRD pattern), was also reported for Sb films (with $d\sim 200$ nm) deposited on (111)Si substrates at room temperature using vacuum thermal evaporation^46^. Such reorientation is even more prominent for thin Sb layers^45^. Thus, the formation of (003)-oriented Sb grains can be a result of low $T_{sub}$ values, or the presence of native Si oxide on the substrate surface. However, the elucidation of underlying formation mechanism of Sb-rich buffer sublayer with oriented grains requires further studies.

**Figure 3:**
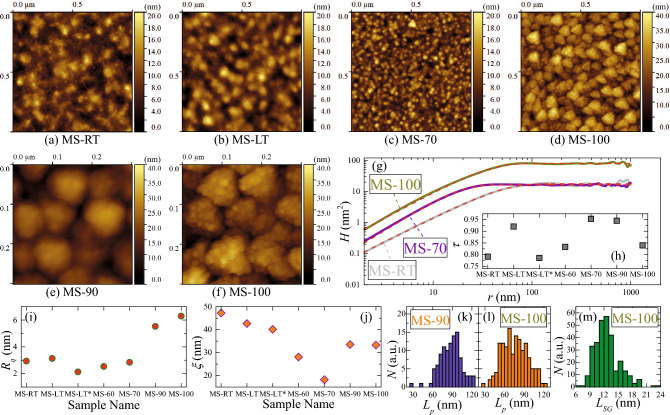
(**a**–**d**) AFM height images of 1 μm × 1 μm surface region for studied samples. Enlarged AFM height images for samples (**e**) MS-90 and (**f**) MS-100. Presented images were processed using Gwyddion software (ver. 2.58). (**g**) Typical HHCF profiles for studied films in double logarithmic scale. Experimental data was fitted using the Eq.(2), fitting curves are shown as red dashed lines. Derived values of (**h**) scaling exponent, $\tau$, (**i**) RMS roughness, $R_q$, and (**j**) correlation length, $\xi$, for studied films. Size distribution histogram for polymer globules in samples (**k**) MS-90 and (**l**) MS-100. Provided $L_p$ values were calculated as the equivalent disc diameters. (m) Linear size distribution histogram for small grains observed on the surface of polymer globules for sample MS-100.

Surface morphology of the films was investigated by atomic-force microscopy (AFM). Typical AFM height images are shown in Fig. 3a–d. Surface morphology of the reference MS-RT sample (Fig. 3a) is rather typical for disordered films. Decreasing $T_{sub}$ and adding weak monomer flow (low $T_{\mathrm {PX}}$) during the deposition process do not change surface morphology of the films qualitatively, as on can see from Fig. 3b,c. However, substantial increase of the monomer flow results in qualitatively different surface morphology (Fig. 3d). Closer inspection of the AFM images for the samples MS-90 (Fig. 3e) and MS-100 (Fig. 3f) reveals the presence of globular-like structures, which is typical for PPX-based films^21,22^. To parametrize the difference of surface morphology for studied samples, we used AFM data to calculate the height-height correlation functions (HHCF): $H(r) = \langle [ h(\vec {x}) -h(\vec {x}-\vec {r}) ]^2 \rangle$ (where $h(\vec {x})$ is the height at a specific point of the image with coordinates $\vec {x}$, and $\vec {r}$ is the displacement vector). The HHCF plots were fitted with the following equation^56^:

2 $$\begin{aligned} H(r)=2R_{q}^2 \left[ 1-\mathrm {exp}\left( -\left( \frac{r}{\xi } \right) ^{2\tau } \right) \right] , \end{aligned}$$

where $R_q$ is the RMS roughness, $\xi$ is the correlation length and $\tau$ is the scaling exponent. Typical HHCF plots and the fitting curves are shown in Fig. 3g (the $R_q$ values are also listed in Table 1). As it shown in Fig. 3h, the $\tau$ values are in the range from 0.75 to 0.95 with no notable correlation with the deposition conditions. One can see from Fig. 3i,j that for the samples without PPX or deposited with low $T_{\mathrm {PX}}$ values there is only small variation of $R_q$, while $\xi$ considerably decreases as the $T_{\mathrm {PX}}$ gets higher. Transition to globular-like morphology at higher $T_{\mathrm {PX}}$ values is accompanied by the abrupt increase of both $R_q$ and $\xi$. As it shown in Fig. 3e,f, there is a slight difference between the samples MS-90 and MS-100. In AFM image of the MS-100 sample one can observe not only polymer globules, but also visible small grains on their surface, which is typical for PPX-based nanocomposite films^21,22^. The size distribution (calculated as the equivalent disc diameters) of polymer globules ($L_p$) in these samples looks rather similar with the mean size of 88 and 75 nm for the samples MS-90 (Fig. 3k) and MS-100 (Fig. 3l), correspondingly, which is close within statistical uncertainty range and also reflected in close $\xi$ values for these films. Size distribution of the small surface grains estimated from the AFM images (Fig. 3d) and shown in Fig. 3m is quite wide with the mean value $L_{SG}$ (diameter) of about 12.5 nm, which is in good agreement with the mean size of MnSb crystallites $D_{Sch} = 13$ nm estimated from the XRD data for this sample. One can suggest that the surface grains in the MS-90 sample are too small ($D_{Sch} = 8$ nm) to be reliably resolved in AFM images (Fig. 3e). Thus, one can suggest that at higher monomer flow (for $T_{\mathrm {PX}}\ge 90$ $^\circ$C) the PPX–MnSb nanocomposite system is successfully formed.

**Figure 4:**
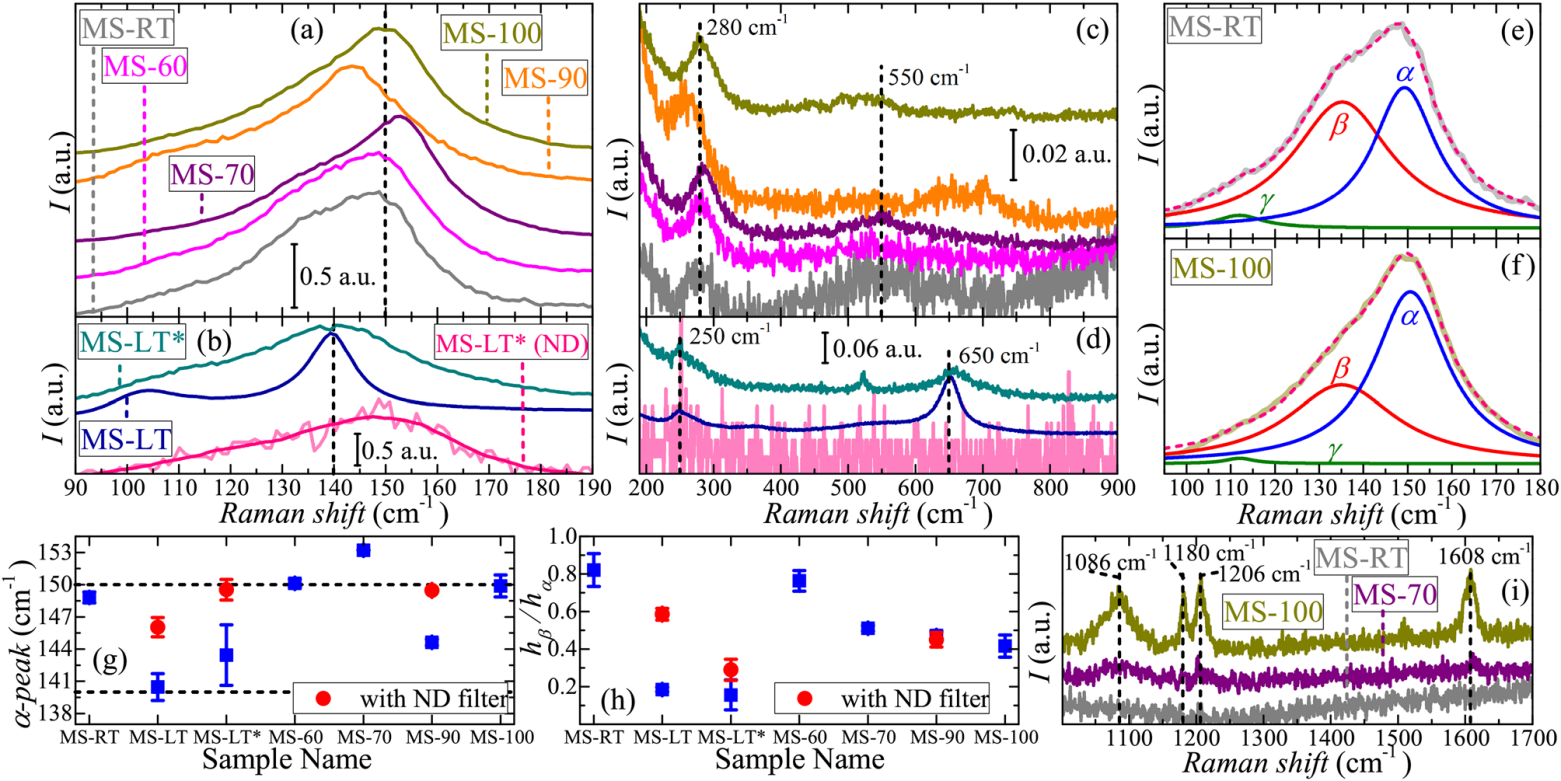
(**a**–**d**) Raman spectra of studied films measured at room temperature, curves are vertically shifted for clarity. Experimental curves in (**a**) and (**c**), (**b**) and (**d**) panels are shown in the same order. Spectra of MS-LT* sample, showed by pink color in (**b**) and (**d**), was recorded using ND filter. Deconvolution of main peak for samples (**e**) MS-RT and (**f**) MS-100, obtained cumulative curves are shown by dashed lines. (**g**) $\alpha$-peak position and (**h**) ratio of $\beta$- and $\alpha$-peaks heights for studied films averaged over several spectra, obtained from different areas of the sample. The results obtained for spectra measured using ND filter are shown by red circles. (**i**) High-frequency parts of Raman spectra for samples MS-RT, MS-70 and MS-100. Black dashed lines in panels (**a**–**d**) and (**i**) show characteristic peak positions in provided experimental spectra.

Figure 4a–d show room-temperature Raman spectra of the samples. All the spectra contain an intensive peak in the 90–190 cm$^{-1}$ range and several weak features in the 200–900 cm$^{-1}$ range. Based on these data the samples can be nominally divided into two subgroups. The position of main peak for the first subgroup of the samples (Fig. 4a) is near 150 cm$^{-1}$, while for the second subgroup it appears to be closer to 140 cm$^{-1}$ (Fig. 4b). Similar difference can be seen in the position of the second peak at 280 cm$^{-1}$ and 250 cm$^{-1}$, correspondingly (Fig. 4c,d). But the most distinguishable difference is that for the first subgroup one can see only a weak peak near 550 cm$^{-1}$, while for the samples MS-LT and MS-LT* there is a well-pronounced peak at 650 cm$^{-1}$. It should be noted that the MS-90 sample shows somewhat intermediate behaviour. During the experiment we observed considerable degradation of studied films under laser radiation. We performed additional measurements using a neutral density (ND) filter that decreased the intensity of incident laser radiation by an order of magnitude. Corresponding spectra have substantially higher noise level (as it can be seen from Fig. 4b,d), so one can reliably distinguish only the main peak and traces of the second feature in the 220–300 cm$^{-1}$ range. Thus, one can assume that the small shift of peak positions and the appearance of weak peak at 650 cm$^{-1}$ can be a result of sample transformation under intensive laser radiation. On the other hand, the observed main peak is characteristic for MnSb inclusions in various composite systems^57–59^. It can be presented as a sum of three peaks^57^, which we noted as $\alpha$-, $\beta$- and $\gamma$-peaks. An example of such representation is shown in Fig. 4e,f. The results of this procedure were averaged over several spectra collected from different areas of each sample. The $\gamma$-peak is typically weak and centered around 110 cm$^{-1}$ and the intensity of $\beta$-peak (around 130 cm$^{-1}$) is substantially varied among the samples. As it shown in Fig. 4g, for the second subgroup of samples the $\alpha$-peak position is closer to 140 cm$^{-1}$, than to 150 cm$^{-1}$ as it is for the first subgroup. However, in the Raman spectra obtained for the second subgroup of samples using ND filter the $\alpha$-peak position shifts towards 150 cm$^{-1}$, while the ratio of $\beta$- and $\alpha$-peaks heights, in average, also becomes closer to the values of the first subgroup (Fig. 4h), as compared to the Raman spectra collected without ND filter. In earlier studies of GaMnSb composites^60^ the $\alpha$- and $\gamma$-like peaks (observed at 152 and 115 cm$^{-1}$) were attributed to Mn-related local modes. However, these two peaks also coincide with the $A_{1g}$ and $E_g$ modes of pure Sb^61^. Thus, only the observation of $\beta$-peak can actually signify the presence of MnSb phase in studied films^57^. The second peak in the experimental spectra (at 250–280 cm$^{-1}$) may have more complex nature, as both, MnSb^57,60^ and pure Sb^62^, can have weak peaks in this region. Lastly, the peaks at around 550 and 650 cm$^{-1}$ can be related to the formation of surface Mn-oxide phases, e.g. MnO$_2$ or Mn$_3$O$_4$, which should yield Raman peaks at 570 and 650 cm$^{-1}$, correspondingly, or their superposition^63,64^. It should be noted that the peak at 650 cm$^{-1}$ was observed in GaMnSb composites with MnSb and Mn$_2$Sb inclusions^58^, and it appears to be more intensive in the sample with higher crystallinity. To the best of our knowledge, the positions of Raman peaks in pure Mn$_2$Sb have not been reliably identified so far. The samples MS-LT, MS-LT* and MS-90 also show the traces of Mn$_2$Sb phase (Fig. 1a), which become less prominent in that order (so does the peak near 650 cm$^{-1}$). Thus, one can to assume that Mn$_2$Sb either have peak at 650 cm$^{-1}$ (i.e. at the same position as the Mn$_3$O$_4$ peak), or the presence of Mn$_2$Sb affects the oxidation processes under strong laser radiation, favoring formation of Mn$_3$O$_4$ over MnO$_2$. Aside from that difference, all studied samples reveal similar Raman features, thus, verifying the presence of MnSb crystallites in studied samples, in accordance with the XRD data. For several samples, including MS-100, additional Raman spectra in the broader range of Raman shifts were recorded (Fig. 4i). As one can see, the sample MS-100 clearly demonstrates additional features, absent for the MS-RT film. Positions of these peaks are in good agreement with literature data^65^ and correspond to PPX. Moreover, the same peaks, but with much smaller intensities, can be distinguished for the MS-70 film, which also suggest the presence of polymer in this sample, in accordance with the XRR results.

**Figure 5:**
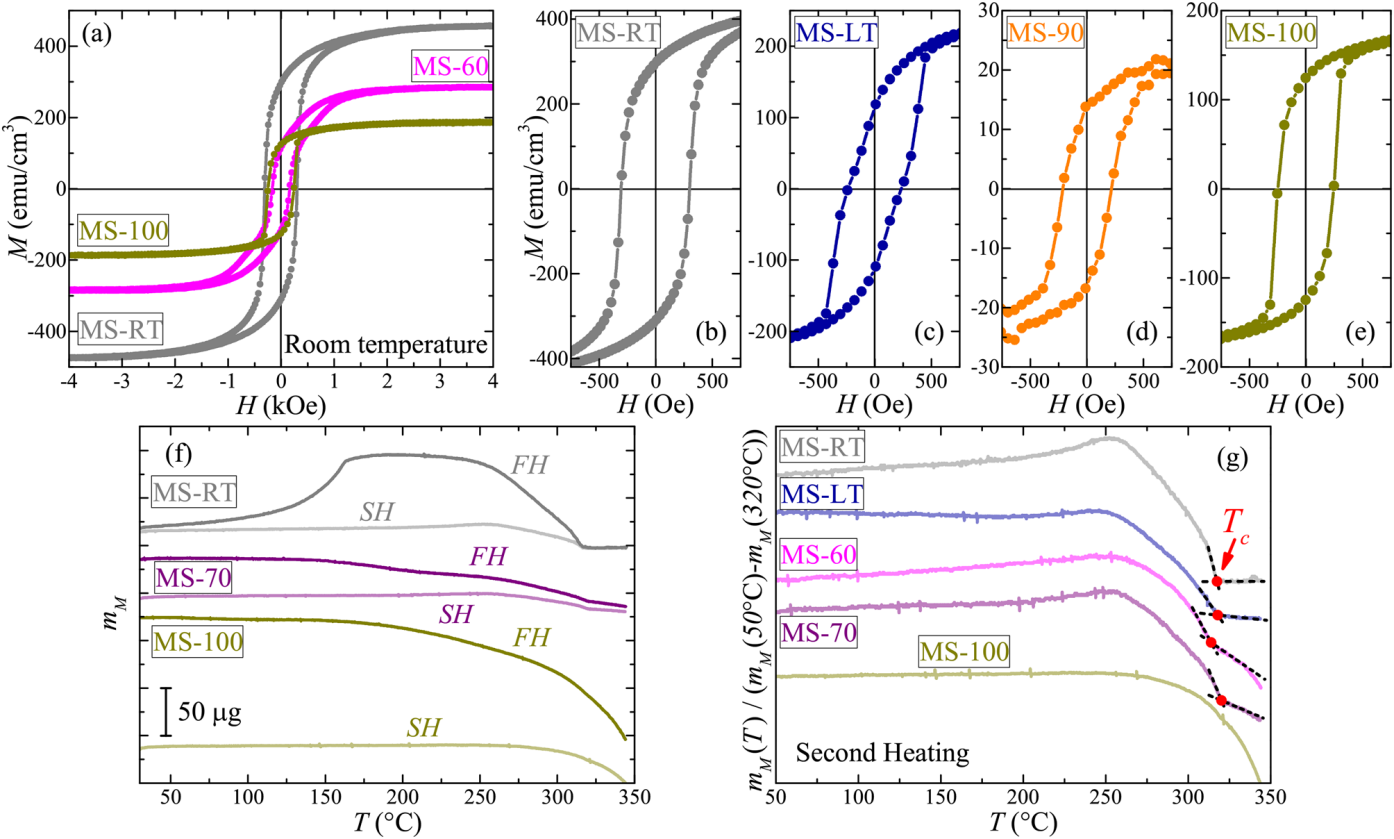
Room temperature (**a**) full magnetization curves and (**b**–**e**) low field regions of corresponding curves for several studied samples. (**f**) TGA curves measured with attached external magnet (at a heating rate of 10 $^\circ$C/min). FH—first heating, SH—second heating. The curves are vertically shifted (in pairs) for clarity. (**g**) Normalized TGA curves measured with attached external magnet during the second heating. The curves are vertically shifted for clarity. Estimated $T_c$ values are marked by red circles.

Magnetic properties of the reference MnSb and PPX–MnSb films were investigated at room temperature using a vibrating sample magnetometer (VSM). All studied films revealed detectable ferromagnetic (FM) response. Typical magnetization curves with subtracted linear background are shown in Fig. 5a. As one can see presented curves show well-pronounced FM hysteresis. However, the shape of hysteresis loop slightly varies among the samples (Fig. 5b–e). Obtained parameters of magnetization curves for the samples (namely, coercive force $H_c$, remanent magnetization $M_{R}$, saturation magnetization $M_{S}$ and corresponding $M_{R}/M_{S}$ ratio) are listed in Table 1. The variation of $M_{S}$ and $M_{R}$ values among studied samples is shown in the Fig. S1a (see Supplementary material). Unlike absolute magnetization values, the $H_c$ values should not be directly affected by the variation of MnSb content. However, as it can be seen in Fig. S1b (see Supplementary material), there is no strict correlation with the mean size of MnSb crystallites. It should be noted that the surface oxidation of MnSb nanoparticles, in principle, may affect the $H_c$ value of a system^30^. This effect should be correlated with the nanoparticle size, however, close $H_c$ values for samples MS-90 and MS-100 (with considerably different $D_{Sch}$ values) suggest that volume oxidation processes may be suppressed in studied films. Basically, the MS-RT sample shows the highest $M_{S}$ and $H_c$ values, while the decrease of $T_{sub}$ and incorporation of polymer lead to the decrease of the corresponding parameters. It should be noted, that previous studies of 30-nm MnSb nanoparticles reported $M_{S}\approx 400$ emu/cm$^{3}$^50^, which is lower than the bulk MnSb value (about 700 emu/cm$^{3}$^33,34^), but close to that for the MS-RT sample with similar mean crystallite size, while the $H_c$ value in our case is 1.5 times smaller than the one reported earlier^50^. For the sample MS-LT* we observed the lowest $H_c$ and $M_{R}/M_{S}$ values, while the rest of studied samples demonstrate closer $H_c$ values, in the range of 160–250 Oe, and $M_{R}/M_{S}$ ratio is in the range of 0.41–0.76. In case of the MS-LT* sample, considerable decrease of FM parameters can be related to increased Sb segregation. Low $M_{S}$ and $M_{R}$ values for the sample MS-90 are, most likely, related to substantial decrease of mean MnSb crystallite size, as it was suggested by the XRD results. Using $M_{S}$ value for the MS-RT sample as a baseline, one can roughly estimate the MnSb content in the MS-100 sample, which have rather close $H_c$ value. Corresponding estimation yields MnSb content of about 40 vol$\%$, which represent a maximum value. However, as it can be seen from the corresponding AFM image (Fig. 3f), such high MnSb content in the sample MS-100 suggests that either the MnSb crystallites are located not only on the surface of polymer globules but also inside them, or $M_{S}$ of each nanoinclusion is substantially higher than that of the crystallites in the MS-RT sample. It should be noted that bulk Mn$_2$Sb is ferrimagnetic with $T_c\approx 277$ °C^66^. Recent experimental studies suggest that pristine Mn$_2$Sb crystals at room temperature should have $M_{S}\approx$ 140–175 emu/cm$^{3}$, $H_{c}\approx$ 100–150 Oe and $M_{R}/M_{S}\approx$ 0.16–0.2^67,68^. Comparing to our results, one can see that even for the samples MS-LT and MS-LT*, with detectable traces of Mn$_2$Sb phase, the variation of FM parameters cannot be consistently related to the contribution of Mn$_2$Sb crystallites, suggesting that primary magnetic properties of studied films are related almost solely to the MnSb nanoinclusions. However, the overall variation of FM parameters suggests that there are additional factors, besides the size-effect and polymer content, that affect magnetic properties of studied films. As it was mentioned earlier the elucidation of such factors for MnSb-based systems is very problematic, as it was suggested by rather controversial results of various groups. One of the possible reasons can be deviation from stoichiometric composition of MnSb crystallites, which should also yield considerable variation of $T_c$ values^31,32^.

To investigate the variation of $T_c$ values we employed thermogravimetric analysis (TGA) measurements performed in the external magnetic field. Basically, after subtracting the sample weight (which was typically around 30 mg) we attached external magnet, which generated fictitious mass $m_M$ around 20–40 μg. We measured temperature dependence of $m_M$ during the first (FH) and the second (SH) heating cycles of the sample, typical TGA curves are shown in Fig. 5f. As one can see, there is a considerable difference between $m_M (T)$ curves, measured during the first and the second heating cycles. For the samples MS-RT and MS-LT we observed substantial increase of $m_M$ during the first heating, which is almost negligible at second heating. For these samples low and high temperature parts of FH and SH curves coincide, suggesting that mass loss during the measurements is small. It should be noted that for thin MnSb film ($\sim 50$ nm) the heating up to $T\approx 400$ $^\circ$C may result in the total loss of FM response due to thermal degradation of MnSb^30^. As we studied relatively thick MnSb films, the absence of such degradation agrees well with the results of other groups^30^. However, for the PPX–MnSb films we observed considerable shift between the FH and SH curves (Fig. 5f), which is roughly proportional to nominal PPX content. Such difference can be related to the thermal destruction of PPX in the composite and evaporation of glue residue (used to fix substrates within growth chamber). Which is why we cannot conclude if the thermal degradation of MnSb upon heating occurs in the PPX–MnSb films. Above $T=250$ $^\circ$C in the FH and SH curves we observed abrupt decrease of $m_M$, which is related to the transition from FM to paramagnetic state. Corresponding part of the TGA curve can be used to estimate the $T_c$ value (as an endset point of step-like decrease) as shown in Fig. 5g for the samples with low PPX content. For the MS-100 sample, with the highest PPX content, we did not observed corresponding feature, which can be simply blurred by the overall mass loss during the heating procedure. Nonetheless, for the MS-RT, MS-LT, MS-60 and MS-70 samples, which have substantially different $M_{S}$ and $H_c$ values, we observed almost constant $T_c$ values of around 314–320 $^\circ$C, suggesting the absence of any considerable deviation from stoichiometric composition of MnSb crystallites in studied films. Therefore, the composition variation may be ruled out as a possible reason of the observed differences in the FM response of studied films. It should be noted, that the heating cycle during the TGA measurements lasted around 30 min and it can be considered as a post-growth annealing. However, the annealing of PPX-based nanocomposites typically results in the aggregation of filler inclusions and relaxation of their crystal structure^21,22,24^, while the variation of inclusions composition during this process seems to be unlikely. It should also be mentioned that the TGA curves do not show any distinguishable peculiarities at $T=277$ $^\circ$C, which confirms secondary role of Mn$_2$Sb phase in the formation of magnetic response of studied films.

Thus, presented results show that a room-temperature ferromagnetic hybrid nanocomposite can be obtained by low-temperature VDP method. Magnetic properties of such films are determined by the MnSb crystallites, while traceable amounts of secondary Mn$_2$Sb phase may affect the shape of Raman spectra recorded under strong laser radiation. It should be noted that the variation of PPX–MnSb films thickness is not proportional to the nominal intensity of the monomer flow, suggesting that the growth mechanism (due to different sticking coefficients of each component) may be different at high and low monomer flow intensities. The parameters of FM response of the films under study are comparable with those reported for MnSb films^42,43^ and nanoparticles^50–52^. We assume that the main factors that negatively affect the FM properties of investigated systems are the decrease of crystallinity and size of MnSb inclusions. On the other hand, the Sb-rich sublayer with oriented Sb grains observed for several samples seems to have no pronounced effect on the FM response. However, in terms of electrical measurements such sublayer is undesirable, because it can shunt the rest of the film (even disordered Sb can retain semimetallic conductivity). Therefore, in future studies one needs to optimize the synthesis regimes in order to avoid formation of such sublayer. Further optimization of synthesis regimes of PPX–MnSb nanocomposite films should be focused on the controlling distances between relatively large MnSb inclusions, in order to retain room-temperature FM phase and to obtain systems with various regimes of conductivity (down to hopping transport regime). Such optimization would specify the perspectives of PPX–MnSb application in spintronic, memrisitve and field-sensing devices.

## Conclusions

We have studied the PPX–MnSb films with various polymer content, synthesized using original VDP method, and the reference MnSb films deposited at various conditions. To the best of our knowledge, the PPX–MnSb nanocomposites are the first hybrid systems with MnSb considered in literature. XRD results verify the presence of MnSb phase in all studied samples, with occasional traces of Mn$_2$Sb. Decreasing the substrate temperature down to − 196 °C and introducing the monomer flow results in the reduction of crystallinity and mean crystallite size for the MnSb phase in studied films. Chosen deposition conditions favor the formation of Sb-rich sublayer with well-oriented Sb crystallites near the substrate, which was confirmed by the results of TEM studies. This sublayer serves as a buffer on which the MnSb or PPX–MnSb layers are grown. Most of the samples show homogeneous microstructure, which is suggested by the SEM and XRR results. The latter also signifies the incorporation of polymer into the films obtained even at low intensities of monomer flow. The increase of monomer flow intensity results in qualitative change of surface morphology of the films, and at high monomer flow the PPX–MnSb nanocomposite is formed. Raman spectroscopy also reveals the presence of MnSb crystallites in all studied films and the incorporation of PPX. For samples with traces of Mn$_2$Sb phase we observed some specific features in the Raman spectra, which can be associated with an effect of strong laser radiation. These results suggest that either the Mn$_2$Sb compound demonstrate Raman peak at the same position as the Mn$_3$O$_4$, or traces of this phase affect the dynamics of Mn oxidation under strong laser radiation. All studied films demonstrate FM behaviour at room temperature. Evaluated $M_{S}$ and $H_c$ values for the investigated samples are comparable to those previously reported for MnSb films and nanoparticles by other groups, but they do not show any simple correlation with basic sample parameters (e.g. nominal composition, presented crystal phases or surface morphology), implying that the exact FM response of studied systems is determined not only by the size-effect or PPX content variation. However, presented data suggest that the Mn$_2$Sb fraction plays almost negligible role in this matter, while the effects of composition variation in MnSb inclusions can be ruled out. The latter stems from the fact that estimated $T_c$ values for studied films are very close to each other as well as to the value typical for bulk MnSb, despite considerable differences in $M_{S}$ and $H_c$ values, which contradicts to the established picture of composition variation in MnSb compound. Although further studies are required to elaborate optimal regimes for synthesis of PPX–MnSb nanocomposites suitable for spintronic and memristive device applications, the obtained results clearly shows that the employment of VDP method is a promising direction of such work.

## Methods

Studied films were obtained by the VDP technique, which includes two basic steps performed in a high vacuum ($10^{-5}$–$10^{-6}$ Torr) chamber. The details of process and scheme of the chamber were described elsewhere^27^. The first step is the co-condensation of monomer (*p*-xylylene–PX) and filler (MnSb) vapors on a substrate, cooled by liquid nitrogen. The PX monomer was prepared by pyrolysis of cyclic dimer of PX ([2.2]*p*-cyclophane) (Daisan Kasei, Japan) at 650 $^\circ$C using classical Gorham’s method^69^. As a filler precursor, we used polycrystalline MnSb powder synthesized via direct fuzing of high-purity elements (Mn 99.8$\%$ and Sb 99.9999$\%$). Single phase of this powder was verified using XRD measurements (not presented here). Filler vapor was produced by thermal evaporation from a tantalum boat. The values of heater current, $I_{\mathrm {MnSb}}$, was deduced basing on the results of preliminary deposition experiments performed with various $I_{\mathrm {MnSb}}$ values, without the monomer flow. Reported value of 13 A corresponds to the lower boundary of observable film formation in these experiments. After each sample synthesis process, the MnSb precursor in tantalum boat was in a form of sintered powder suggesting that the melting of MnSb occurred primarily at powder grain boundaries. The latter implies that the actual temperature of tantalum boat during the synthesis process was lower than the melting temperature of MnSb (840 $^\circ$C). The second step of VDP process is the slow heating of the obtained co-condensate up to room temperature, during which the polymerization of PX into PPX should occur, as well as the formation of filler nanoinclusions. At low PX flow rates (i.e. for low $T_{\mathrm {PX}}$ values) the inverse composite may occur—PPX inclusions in the inorganic matrix. Reference films (obtained with zero PX flow) were deposited on substrates either at room or liquid nitrogen temperature, with consequent slow heating up to room temperature. As substrates we used (100)-oriented Si plates with thickness of 0.6 mm.

XRD and XRR studies were performed on a Rigaku SmartLab diffractometer (Rigaku, Japan) with a rotating Cu-anode (CuK$\alpha _1$ radiation, $\lambda _{X} = 1.5406$ Å). A multilayer parabolic mirror and a Ge(220)x2 monochromator were used to collimate the incident beam. Obtained XRD patterns were processed using ICDD PDF-2 powder references. The approximation of XRR curves was performed to obtain smoothed (with allowance for the transition layers) electron density profile. The profile is divided into thin sublayers, within which the change in the electron density can be neglected. The theoretical XRR curves were calculated for the initial system of sublayers (which utilized basic parameters of studied films) based on the Parratt recursion formalism^70^, and then the parameters of the initial electron density profile were refined by minimizing the discrepancy between the calculated and experimental data.

The thickness of the films was estimated using dual beam scanning electron microscope/focused ion beam (SEM/FIB) Versa 3D (FEI, USA), equipped with a Schottky field emission gun. To obtain clear film edge, samples were locally (approximately 10 μm × 10 μm area) covered with a 2 μm W protective layer and etched using Ga$^+$ FIB. The tilt of the film edge in the obtained images was taken into account in thickness determination. Investigated films were also studied using the scanning/transmission electron microscope (S/TEM) Titan 80-300 (FEI, USA) operated at 300 kV and equipped with the spherical aberration (Cs) corrector (electron probe corrector), high-angle annular dark-field detector (HAADF) (Fischione, USA) and GIF Tridiem post-column energy filter (Gatan, USA). Digital micrograph software (Gatan, USA) and TEM Imaging&Analysis software (ThermoFisher Scientific, USA) were used for the image processing. The films cross-sections for the TEM study were prepared by the standard procedure that included mechanical polishing of strips of the samples glued “face-to-face” down to a thickness of 20–25 μm followed by ion milling (Ar$^+$) using the Gatan 691 Precision Ion Polishing System (Gatan, USA) at an accelerating voltage of $U =3$ kV until perforation. The final milling was carried out by Ar$^+$ ions with energy of 0.1 keV. The overall procedure of sample preparation and TEM studies was similar to that described earlier^71^.

Surface morphology of the films was investigated by atomic force microscopy (AFM) in PeakForce Tapping mode using a Multimode 8 microscope with a Nanoscope V controller (Bruker, USA). Triangular silicon nitride cantilevers with silicon tips ScanAsyst-Air (Bruker, USA) having nominal force constant of 0.4 N m$^{-1}$ and resonance frequency of about 70 kHz were used as AFM probes. All images were recorded in air at room temperature. Obtained AFM images were processed using Gwyddion and ImageJ software.

Raman spectra of studied samples were recorded in air at room temperature using a NTEGRA Spectra system (NT-MDT, Russia). Presented Raman spectra were recorded in the backscattering geometry using 488 nm laser with nominal maximum power of 100 mW and typical exposition time of 60 s. For several samples additional spectra were recorded using neutral density (ND) filter, which decreased the intensity of incident laser radiation by an order of magnitude. The samples MS-RT, MS-70 and MS-100 were additionally studied in a broader range of Raman shifts.

Magnetic properties of the synthesized films were investigated using a vibrating sample magnetometer 7400 (Lake Shore Cryotronics, USA). Experiments were carried out in magnetic field up to 5 kOe at room temperature, magnetic field was oriented in the sample plane. Additional measurements of empty sample holder were performed in between samples measurements to rule out possible parasitic contributions.

Data of thermogravimetric analysis were recorded in N$_2$ atmosphere (with 100 ml/min flow) using a Pyris 1 TGA (PerkinElmer, USA) analyzer. After initial weighing of studied sample the external magnet was attached, which generated fictitious mass due to attraction of sample to magnet (which allowed us to estimate $T_c$ as an endset point of step-like decrease upon heating the sample). Analogous procedure is used for TGA temperature calibration (by measuring the Ni reference with reproducible value of $T_c$). Presented TGA curves were recorded at a heating rate of 10 °C/min in dynamic mode. After first heating stage the heater was turned off and the sample was cooled down to room temperature in nitrogen atmosphere. After this the second heating stage was performed.

## Supplementary Information

Supplementary Information.
