# Supplementary material for: Structural characterization and magnetic response of poly(p-xylylene)–MnSb and MnSb films deposited at cryogenic temperature
Source: Sci Rep. 2021 Aug 6;11:16004. doi: 10.1038/s41598-021-95475-9 (PMC8346481; doi:10.1038/s41598-021-95475-9)
Supplement: Supplementary file 1 — Supplementary Information. [file 41598_2021_95475_MOESM1_ESM.pdf]

Supplementary material: Structural characterization and magnetic response of poly(p-xylylene)–MnSb and MnSb films deposited at cryogenic temperature

L.N. Oveshnikov[1,*], S.A. Zav'yalov[1], I.N. Trunkin[1], D.R. Streltsov[1,2], N.K. Chumakov[1], P.V. Dmitryakov[1], G.V. Prutskov[1], O.A. Kondratev[1], A.A. Nesmelov[1], and S.N. Chvalun[1,2]

[1]National Research Center "Kurchatov Institute", Moscow, 123182 Russia
[2]N.S. Enikolopov Institute of Synthetic Polymeric Materials, RAS, Moscow, 117393 Russia
[*]oveshln@gmail.com

1 Additional illustrations

Fig. S1 visualize the data given in Table 1 of the main text. Fig. S1a shows variation of remanent (M_R) and saturation (M_S) magnetization values of studied samples at room temperature. Data for samples MS-LT, MS-60, MS-70, MS-90 and MS-100 represents the effect of polymer content increase on magnetization values of PPX–MnSb films. The difference between samples MS-LT and MS-LT* is that the latter was obtained at higher rates of MnSb precursor evaporation (higher heater current value). Fig. S1b shows the coercive force (H_c) for studied films as a function of mean size of MnSb crystallites, estimated from XRD data presented in the main text.

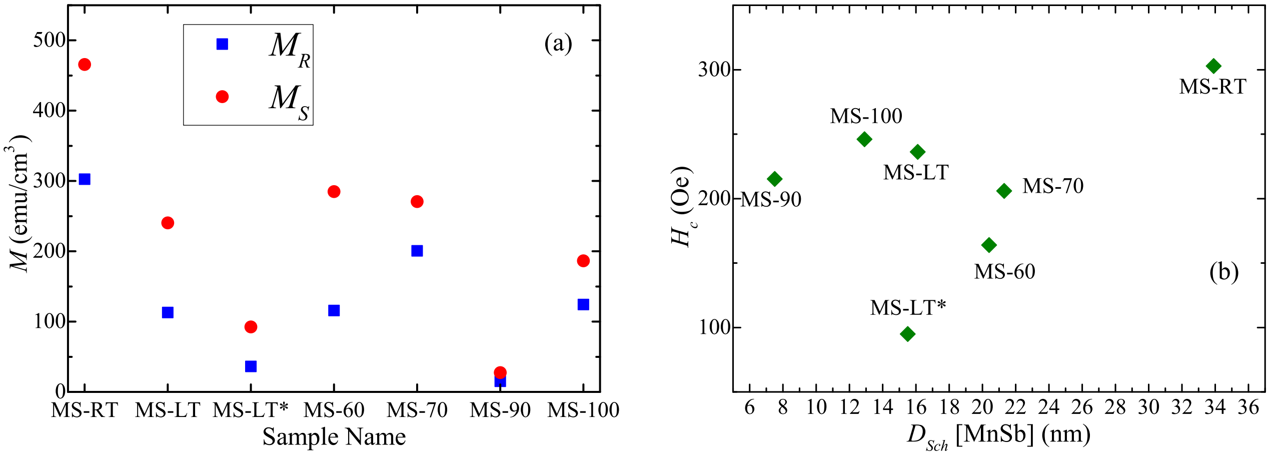

Figure S1. (a) Remanent and saturation magnetization values of studied films. (b) Coercive force of the films as a function of mean size of MnSb crystallites, estimated from XRD data.
